# Internationalized at work and localistic at home: The ‘split’ Europeanization behind Brexit

**DOI:** 10.1111/pirs.12350

**Published:** 2017-12-27

**Authors:** Riccardo Crescenzi, Marco Di Cataldo, Alessandra Faggian

**Affiliations:** ^1^ London School of Economics Geography and Environment Houghton Street London WC2A 2AE United Kingdom; ^2^ Gran Sasso Science Institute, Social Sciences L'Aquila Italy

**Keywords:** Brexit, voting, FDI, trade, UK, European Union

## Abstract

This paper looks at the results of the referendum on the United Kingdom membership to the European Union in order to test the link between the internationalization of the local economy and the openness of the local society as factors associated with the Leave vote (Brexit). The paper compares a number of alternative explanations put forward in the public debate after the referendum. The empirical analysis suggests that the outcome of the referendum can be linked to an increasing tension between the ever increasing internationalization of local firms and the ‘localistic’ attitude of their employees. Brexit can be seen as the result of a process of ‘split Europeanization’ whereby Euroscepticism is triggered by the increasing mismatch between internationalized economies (and corporate economic interests) and localistic societies (and workers’ attitudes and cultural preferences).

## Resumen

Este artículo analiza los resultados del referéndum sobre la membresía del Reino Unido a la Unión Europea con el fin de poner a prueba el vínculo entre la internacionalización de la economía local y la apertura de la sociedad local como factores asociados con el voto a favor de Salir (Brexit). El artículo compara varias explicaciones alternativas presentadas en el debate público después del referéndum. El análisis empírico sugiere que el resultado del referéndum puede vincularse con una tensión creciente entre la internacionalización cada vez mayor de las empresas locales y la actitud ‘localista’ de sus empleados. Brexit se puede entender como el resultado de un proceso de ‘europeización dividida’ mediante el cual el euroescepticismo se desencadena a causa del creciente desajuste entre las economías internacionalizadas (con intereses económicos corporativos) y las sociedades localistas (con actitudes y preferencias culturales de los trabajadores).

## 抄録

本稿では、イギリスの欧州連合離脱の是非を問う国民投票の結果を検討し、地方経済の国際化と地方社会の開放性の関連が、離脱票[ブレグジッド(Brexit:イギリスのEU離脱)]につながる要因であったか否かを検証する。ここでは国民投票後の公開討論で提示された、投票結果に関する多数の仮説を比較する。実証分析から、国民投票の結果は、国際化傾向を続ける地方企業と「地方主義的志向(localistic attitude)」のその企業の従業員との緊張状態の悪化が関連していることが示唆される。国際化した経済(および企業の経済的利益)と地方主義的社会(および労働者の志向と文化的嗜好)のミスマッチの拡大により起きた欧州懐疑主義の観点からみると、ブレグジッドはヨーロッパ統合の分断のプロセスで起きた結果であるととらえることができる。

## 1 INTRODUCTION

23 June 2016 will always be remembered as a date which sent shockwaves not only throughout the United Kingdom but throughout Europe and the world. The referendum on the UK membership to the European Union delivered a largely unexpected result, starting the process that will (presumably) lead to the departure of the UK from the EU (also known as “Brexit”) in March 2019. Following this historical turning point, analysts and scholars around the world, started asking themselves two key questions: (*i*) why did this happen? and (*ii*) what will be the likely consequences of this event? Answering either question in full constitutes an enormous challenge for social scientists. Before addressing the latter question beyond speculation, we will need to wait until the consequences of the actual UK departure from the EU will unfold over time. Conversely, it is already possible (and much needed) to address the former question by looking at some key facts and figures on the social, economic and behavioural conditions that influenced voting patterns and shed new light on the underlying ‘dis‐integration’ forces that might, in different shapes or forms, affect other EU countries. This paper aims to contribute to this endeavour by focusing on the Brexit referendum results and linking them with the internationalization and openness of the local economic and social environment of the UK regions.

In the days and months following the Brexit vote, several hypotheses have been put forth on the reasons behind the referendum results. Commentators have pointed their fingers towards demographic variables (such as age and education) or towards a response to increasing immigration and pressures on social services. Although it is now apparent that the Brexit vote was indeed influenced by age and education factors – with younger and better educated voters choosing to remain in the EU – other explanations have more limited support in simple descriptive data. Indeed, high‐immigration constituencies were more likely to vote in favour of continued EU membership. Conversely, many areas receiving high shares of EU funds – a relevant source of public spending in deprived regions – overwhelmingly supported Brexit. The multiplicity of factors influencing Brexit votes and the lack of explanatory power of individual variables call for more nuanced explanations.

This paper aims to give a more thorough picture of the localized factors behind the Brexit vote by testing the hypothesis that one of the reasons behind the outcome of the Brexit Referendum is the inherent tension between the ‘internationalization of production’ and the ‘(lack of) globalization of society’. While UK firms (including those located in areas where the Leave vote prevailed) are highly globally inter‐connected, the same is not true for the local workforce that lacks in non‐local social connections and cultural ties. This fundamental tension between the internationalization of production and the localistic social environment of many areas might be a stronger predictor for anti‐EU votes, reflecting a more fundamental fracture in the process of European integration (“split Europeanization”) between Europeanized regional economies and Eurosceptic societies.

Together with the many non‐academic pieces looking at various correlations between the percentage of Leave voters and factors such as age, education, political affiliation, presence of migrants and so on, a growing number of academic papers have attempted to give a more comprehensive and multifaceted view of the reasons motivating the Brexit vote, among which the most notable contributions are Arnorsson and Zoega (2016), Becker, Fetzer, and Novy (2016), Goodwin and Heath (2016), Harris and Charlton (2017), and Manley, Jones, and Johnston (2017). However, the innovative contribution of our paper is twofold. First, we model more explicitly the role of firms’ internationalization by using inward and outward FDI as well as the Los, McCann, Springford, and Thissen's (2017) index of regional exposure to EU trade. Second, we explicitly compare economic internationalization with local social openness as a key explanation for the Referendum results.

The organization of the paper is as follows. Section 2 describes the status quo of the research on the drivers of the Brexit vote. Section 3 describes the simple empirical exercise, followed by a description of the data. Section 4 presents the results and discusses them. Finally, Section 5 concludes and provides some reflections for EU policy actions.

## 2 WHAT WE KNOW (OR DO WE?) ABOUT THE CAUSES OF BREXIT

Some of the key questions floating around in the days after Brexit were: why did this happen? Who are the ‘Brexiters’? All national and international newspapers provided several interpretations of the phenomenon pointing at some common characteristics of the voters who opted for the Leave option. It became clear that the electorate was split according to demographic characteristics, most importantly age and education. The percentage of voters in favour of Brexit increased monotonically with age, going from only 27% of the group aged 18‐24 to 60% of those aged 65+, 1
http://www.bbc.com/news/uk‐politics‐36616028. while, conversely it decreased monotonically with education. The main story that was told was one of cultural values, with an insurmountable generational gap between the younger generation (more “Euro‐philes”) and the older generation more attached to traditional British values and more xenophobic (see Burn‐Murdoch, 2016 among others).

The emphasis on cultural priors and values – all factors hard to affect by means of public policies at any level – offers limited insights on how further political tensions towards international disintegration and isolation can be mitigated in the UK and in other EU member states. This approach tends to downplay the importance of economic factors as drivers of voting behaviour at the Referendum. Some authors went as far as to explicitly discard any economic dimension behind the vote, defining Brexit as a purely cultural/psychological phenomenon. 2Such approach, explaining the Brexit outcome exclusively on the basis of cultural motivations, resonates with the literature analysing voters’ attitudes towards the EU and arguing that these are mainly rooted in their cultural identity (e.g. Carey, 2002; McLaren, 2002; Sides & Citrin, 2007). In his commentary whose title reads “It's NOT the economy, stupid: Brexit as a story of personal values”, Kaufmann (2016) claimed that the key factor in voting to leave the EU was identity and that in explaining Brexit “age, education, national identity and ethnicity are more important than income and occupation”. According to Hobolt and Wratil (2016), control over immigration was considered the most important issue for Leave voters, while economic arguments were considered less or not relevant for their voting decision. These views align with the one of other influential commentators, such as Scruton (2016), arguing that, in choosing Leave, UK voters were expressing their attachment to an inward‐looking form of national collective identity – in opposition to the one of those supporting the global, outward‐looking European integration project – and demonstrating their discontent with what they perceived as a deprivation of national sovereignty. Scruton (2016) considered immigration, the EU democratic deficit and the effect of the European courts on the law and customs of the British people as the main factors having influenced the Leave vote. All these arguments resonate with Goodhart's (2017) dichotomy between the ‘anywheres’, that is, a minority of British people, culturally and socially dominant in the country, mobile, highly‐educated, with ‘achieved’ identities, more prone to vote remain, and the ‘somewheres’, namely, the relative majority of UK citizens, older and less well educated, more rooted in their geographical identity, finding the rapid changes of the modern world unsettling, and more inclined towards Leave.

The initial academic contributions analysing the determinants of Brexit have indeed confirmed the role of political, cultural, and demographic elements as fundamental drivers of voting choices. Arnosson and Zoega (2016), Clarke and Whittaker (2016), Harris and Charlton (2017) and Manley et al. (2017) confirmed the ‘age and education effects’ on the probability of voting to leave the EU. Harris and Charlton (2017) also found that having an intermediate or low‐skilled occupation (traditionally associated with lower human capital) significantly increased the chances of having voted for Brexit. Arnosson and Zoega (2016) and Clarke and Whittaker (2016) also emphasized the relevance of immigration in conditioning the Referendum vote, a result in line with Langella and Manning (2016) who identified immigration and demographic factors as key elements behind Brexit. Along similar lines, Hobolt (2016) claimed that favouring Brexit was common among the less‐educated, poorer and older voters, and among those who express concerns about immigration and multi‐culturalism. Finally, Goodwin and Heath (2016) highlighted the close relationship between support for the UK Independence Party (UKIP) – a political party with a clear eurosceptic and anti‐immigration platform 3The UK Independence Party is a “British political party [which] espouses a populist libertarian philosophy centred on the withdrawal of the United Kingdom from the European Union” (Encyclopaedia Britannica accessed in June 2017 at https://www.britannica.com/topic/United‐Kingdom‐Independence‐Party). On the relevance of immigration concerns and euroscepticism among UKIP supporters see Goodwin and Milazzo (2015). – and Leave votes at the Referendum, adding that public support for Brexit was more polarized by education level than support for UKIP has ever been.

However, conceptual perspectives motivating Brexit exclusively on the basis of cultural issues (identity, national sovereignty, etc.) have been regarded by some as inadequate in describing the geography of the Brexit vote in an exhaustive way (Los et al., 2017). This alternative view claims that variables accounting for the economic conditions of citizens and the economic geography of UK regions are at least as important as culture and identity to determine individual attitudes towards the EU and voting patterns at the 2016 Referendum.

Indeed, empirical analyses performing comprehensive investigations of the Brexit vote and considering not only demographic and political variables but also proxy variables for local economic structure and ‘economic exposure’ to the rest of the European Union, all seem to suggest that economic factors have played a significant role. Becker et al. (2016), Arnosson and Zoega (2016) and Hobolt (2016) all provided evidence demonstrating that voters’ decisions have been clearly conditioned by their level of income, with less well‐off citizens and poorer areas more likely to support a Leave vote. Adding to that, Bell and Machin (2016) and Darvas (2016) claimed that wage inequality and poverty are two crucial drivers of Brexit. Clarke, Goodwin, and Whiteley (2017) demonstrated that economic cost‐benefit evaluations are at least as influential as any sense of identity. Indeed, Curtice (2017) claimed that the perceived impact of leaving the EU on the economy is the variable more strongly related to how people voted. Another work accounting for both cultural (nationalistic) attitudes and economic motivations is Colantone and Stanig's (2016) in‐depth analysis of the impact of trade‐related transmission channels from economic globalization into Brexit voting patterns. They suggested that economic integration via trade did impact Eurosceptic preferences. More than the actual incidence of migrants into an area, they argued, it is globalization‐induced shocks that drove perceptions and attitudes towards immigration, and in turn determined voting decisions on Brexit.

Additional evidence on the relevance of socio‐economic factors as Brexit determinants is reported in the study by Clarke and Whittaker (2016), which showed how labour market conditions are crucial in conditioning voters’ choices. Higher employment levels are associated with lower propensity towards Leave, suggestive that unemployed people were more prone towards Brexit than those with safe salaries and jobs.

Even the result of a link between public support for UKIP and a higher propensity to vote Leave can be interpreted in economic terms. While it is true that the UKIP electorate is composed of people feeling culturally excluded, it is also true that support for UKIP is high particularly among those who have been ‘left behind’ by the economic transformation of Britain in the recent decades (Ford & Goodwin, 2014) and that perceive the process of globalization of the world economy as disadvantageous (Clarke, Whiteley, Borges, Sanders, & Stewart, 2016; Goodwin & Heath, 2016; Goodwin & Milazzo, 2015). Hence, cultural and economic motives are not necessarily mutually exclusive, but rather they can be intertwined.

### 2.1 When the ‘internationalization’ of the economy clashes with cultural ‘localism’

The review of the current debates on the factors influencing the outcome of the Referendum suggests that neither economic nor cultural arguments alone are sufficient to provide a complete picture of the voting patterns on Brexit. What this paper aims to put forward is a more nuanced explanation that accounts simultaneously for both economic and cultural aspects that intrinsically co‐exist and interact in any society.

Purely cultural explanations of the Referendum outcome inevitably rely on the strong assumption – as in Goodhart (2017) – that people living in areas such as Bradford, Hull or Sheffield (where Leave prevailed) have more connection, devotion or sense of rootedness to their home towns than strongly pro‐Remain locations such as Manchester, Leeds or Liverpool. Indeed, a large and consolidated body of literature on social ties and social capital has shown that the extent to which individuals are rooted into a local community is not necessarily in contrast with their attachment to other localities and social groups. The way in which individuals combine embeddedness into local networks and connectivity with external groups is highly heterogeneous across places and communities (Granovetter, 1973, 1982, 1985).

Conversely, explanations based on economic motives are hard to reconcile with some key hard facts on the economic geography of the UK and the spatial patterns of the Leave vote. Peripheral areas relying more heavily on employment generated by large foreign corporations have overwhelmingly supported Brexit. As an example, in the area of Sunderland only about a third of people voted Remain despite the presence of a “giant Nissan factory which exports more than half its output to the other EU countries” (Begg, 2016, p. 33). A similar case is North Wales, home of a very large Airbus factory and yet favouring Brexit. All these areas seem to have voted against their own economic self‐interest. A similar ‘paradox’ is highlighted by Los et al. (2017), suggesting that UK regions more economically integrated into the single market, that is, exporting higher shares of their GDP to the EU were more ‘Eurosceptic’ and disproportionately favoured leaving the EU.

Local jobs in many UK regions are highly dependent on foreign economic ties: foreign companies are active in local labour markets with their subsidiaries; domestic companies can preserve employment levels by off‐shoring some of their activities abroad; integration into international trade flows is central to the competitive strategies of a large share of local (foreign and domestic) firms increasingly more embedded into global value chains. Therefore, while ‘internationalized firms’ absorb substantial shares of the regional workforce of many UK regions, workers, managers and entrepreneurs in some of these regions openly rejected internationalization by voting to rescind their links with the EU, the largest integrated market and investment space of the world.

It is precisely by exploring this apparent ‘paradox’ that new insights on the logic of the Brexit vote can be gained. In particular, two fundamental dimensions should be taken into account in order to assess the degree of internationalization of the various regional economies from a new standpoint. First, internationalization is determined not only (and not necessarily) by import and export flows that have attracted the attention of the existing literature on the economic motives of the Brexit Referendum. Domestic workers compete with other workers active in foreign locations via the inflow of imported goods (that can be sold on the local market at lower prices than domestically supplied alternatives thanks to cheaper inputs) but they might also be employed by the local subsidiaries of foreign firms (or by domestic firms with subsidiaries abroad). In other words, both foreign and domestic multinationals (i.e., the degree of active and passive internationalization) connect local and ‘global’ labour markets, introducing different and new work practices and standards, offering the opportunity to work in more internationalized environments, but also fostering competitive pressure on domestic workers.

Second, voters’ decisions are not exclusively influenced by their work‐place dynamics, as advocated by the literature on the cultural factors behind Brexit. Following Granovetter (1973), social ties can be supported by frequent contacts with deep emotional involvement (‘strong ties’) or by sporadic interactions with low emotional commitment (‘weak ties’). ‘Strong ties’ are associated with local connections and communication of familiar information and ideas within like‐minded groups thanks to a set of shared codes, norms and values. Strong ties function as a bonding device within homogeneous groups potentially hampering the degree of sociability outside closed circles. Conversely, weak ties operate as a bridge between otherwise disconnected social groups (Ruef, 2002). Therefore, areas where bonding social capital (Putnam, 2000; Rodriguez‐Pose & Storper, 2006; Storper, 2005) prevails are less likely to develop a positive and synergistic approach with reference to the internationalization of the economy, hampering connections “outside one's own immediate network or social circle and into new areas of information and opportunity” (Cooke, Clifton, & Oleaga, 2005). Workers from ‘high bonding social capital’, culturally localistic and inward‐looking communities are more prone to see employers’ internationalization as a (potential) threat to their economic stability and welfare. These workers are more prone to take any occasion to vote ‘against economic integration’ in order to re‐align the economy to their social preferences. Conversely, workers that are immersed in open and outward‐oriented local societies are more capable to bridge different social groups, understand and embrace a multi‐national working environment, maximize the potential learning benefits from the international division of labour and constructively cope with interaction and competition from foreign workers.

As a result, in order to shed new light on the factors behind the Leave vote, it is necessary to cross‐fertilize the literature on economic motives of the Brexit vote with the literature on its socio‐cultural explanations and test the hypothesis that voters immersed in culturally inward‐looking communities responded to the foreign projection of their employers seen as a threat to their job security and salary growth rather than as an opportunity for development and long‐term prosperity. The Brexit referendum offered a unique opportunity to ‘punish’ more outward‐looking employers and – at the same time – reduce the frightening competition from ‘invisible’ fellow‐workers located in foreign countries exerting a very concrete competitive pressure on local labour markets. Following this line of reasoning the result of the Brexit referendum can be seen as the result of a process of ‘split Europeanization’ whereby Euroscepticism is triggered by the increasing mismatch – in specific constituencies – between internationalized firms (and corporate economic interests) and ‘localistic’ employees (and workers’ attitudes and cultural preferences).

## 3 EMPIRICAL MODEL AND DATA

In order to test the ‘split Europeanization’ hypothesis discussed above, it is possible to estimate a simple local voting equation that predicts the share of Leave votes (i.e., the share of votes to leave the EU in the 2016 Referendum) in the UK NUTS 2 4The NUTS classification system (*Nomenclature des Unités Territoriales Statistiques*) is a statistical subdivision of European regions administered by Eurostat, which has been established with the purpose of collecting comparable data on regional characteristics across time in the EU. The level of NUTS 2 regions has already been employed by previous studies investigating the factors contributing to influence the outcome of the Brexit Referendum (Arnosson and Zoega, 2016; Fidrmuc et al., 2016). regions as a function of a set of their social and economic characteristics that include proxies for the internationalization of both production and society as well as their interaction term. The voting model is specified as a simple linear equation to be estimated with OLS where the percentage of Leave votes in the 2016 EU Referendum by NUTS 2 region *i*. *Leave*_*i*, 2016_ (with *i* = 1,2,...,33 5The UK is composed of 37 NUTS 2 regions. However, for some key variables of interest data is unavailable for Northern Ireland and for the four NUTS 2 Scottish regions. Given that for Scotland as a whole the data are sometimes available our final sample is made of the 32 NUTS 2 regions of England and Wales, plus Scotland.) is the dependent variable and the explanatory variables can be classified as follows.

### 3.1 Internationalization of the economy (FDI and trade)

The first set of variables of interest is linked to the ‘EU exposure’ of the regional economies and their internationalization not only in terms of trade flows but also, and more importantly, in terms of inward and outward FDI flows. Although still in an imperfect and coursed fashion, these variables can capture the insertion of the regional economy into global investment flows. To this aim, the model considers three different variables: the cumulative value of inward FDI from the EU over the 2003–2014 pre‐referendum period (*FDI*_*INi*_) expressed in US Dollars and in number of new jobs created per 1,000 inhabitants; the cumulative value of outward FDI from each region towards the EU in the same period (*FDI*_*OUTi*_); the percentage of exports of an area towards the European Union, as in Los et al. (2017) (*Trade*_*i*_).

### 3.2 Internationalization of society

In order to proxy in a parsimonious way for the degree of internationalization and social openness to foreign cultural linkages of UK regional societies the model relies on two key variables: the percentage of voters for the UK Independence Party (UKIP) in the 2015 elections (*UKIP*_*i*_) and a purpose‐built “Cultural openness index” (*Openness*_*i*_).

The latter index is a composite indicator that linearly combines the “percentage of UK‐born whose native language is English who are capable to converse in another foreign language” 6This indicator does not capture the presence of immigrants or foreigners in the region (this specific aspect is controlled for by means of a dedicated variable in the controls). Conversely, this indicator captures the extent to which ‘native British citizens’ (i.e., voters in the Referendum) are able to converse in other languages showing an interest and a predisposition for other cultures and countries. and “the expenditures per inhabitant for outbound trips”. 7This variable refers to expenditures within the UK by UK residents making an overseas trip (typically money spent at airports or on transport fares). The sources are: UK TSA 2013 (ONS), ONS IO&SUT 2013, Annual Business Survey 2013; GB Day Visits Survey 2013; GB Tourism Survey 2013; International Passenger Survey 2013. These indicators can be considered – when combined – as an overall measure for the cultural internationalization of voters and their international social engagement.

### 3.3 ‘Split Europeanization’

The interaction term between inward FDI (the key proxy for economic internationalization) and the ‘cultural openness index’ will make it possible to directly test the hypothesis that, *ceteris paribus*, it is the tension between internationalized economies and localistic (i.e., low in cultural openness) societies that may have led many British citizens to vote to leave the EU.

Following the emerging literature on the drivers of the Leave vote in the Brexit referendum, the model controls for a set of other possible factors influencing the Referendum result.

### 3.4 Demographic factors

To proxy for age and education, which are presented in the existing work among the most important determinants of the vote we use: the percentage of the population aged 20–34 (*Age*_*i*_) and the percentage of employed people with tertiary education (*Educ*_*i*_).

### 3.5 Economic factors

To control for unemployment (and economic deprivation more generally), we use the percentage of population claiming Job‐Seeker Allowance (JSA) benefits (*U*_*i*_) in the period before the referendum (2008–2014). The two‐year lag between 2014 and the referendum of 2016 helps mitigate possible endogeneity issues.

The existing literature suggests that higher unemployment in an area might result in an anti‐globalization sentiment and resentment against the government (supporting Remain positions), hence increasing the percentage of Leave voters.

### 3.6 Direct transfers from the EU budget

The decision to leave the European Union is highly likely to discontinue EU funding in particular under the Common Agricultural Policy and the EU Structural Funds. Areas in receipt of these funds might be more likely to prefer to remain in the EU in order to keep their current level of funding. For voters in these constituencies the cost to leave the EU is, *ceteris paribus*, going to be higher due the loss of EU subsidies. Therefore, it is important to also control for areas which have been recipients of EU funds (*EU funds*_*i*_), by including in the model the average annual EU structural fund payments in the period before the referendum (2007–2013).

### 3.7 Immigration

As one of the key point used by the Leave campaign was the role played by immigration and the need to control it, we also included a control for growth of immigrants in the area in the period 2001–2011 (*Immig*_*i*_).

### 3.8 Model

Formally, the final model is summarized in Equation 1:

(1) $${\text{Leave}}_{i,2016}=\alpha +\beta_{1}{\mathrm{FDI}}_{\mathrm{INi}}+\beta_{2}\;{\text{Openness}}_{i}+\beta_{3}\left({\mathrm{FDI}}_{\mathrm{INi}}\times {\text{Openess}}_{i}\right)+\beta_{4}\;{\text{UKIP}}_{i}+\beta_{5}\;{\mathrm{Age}}_{i}+\beta_{6}\;{\text{Educ}}_{i}+\beta_{7}\;U_{i}+\beta_{8}\;\mathrm{EU}\;{\text{funds}}_{i}+\beta_{9}\;\text{Immig}+\beta_{10}\;{\text{Trade}}_{i}+\beta_{11}\;{\mathrm{FDI}}_{{\text{OUT}}_{i}}+\varepsilon_{i}.$$

Throughout the analysis standard errors are clustered at the NUTS1 level, corresponding to UK government office regions, in order to mitigate issues of spatial autocorrelation. 8Clustering at NUTS 1 level in our analysis implies relying only on 11 clusters. We select this geographical level of clustering because it is more representative of UK functional economic areas than NUTS 2 regions. As an example, NUTS 1 correspond to the full territory of three UK Home Nations: Wales, Scotland, and Northern Ireland. However, adopting NUTS 2 instead of NUTS 1 geographical units for the clustering leaves the main results of the analysis unchanged (regression results available upon request).


In order to build the database required for the analysis, we combined a large number of different data sources on the economy and society of the UK regions. Table 1 summarizes the variables and their sources.

**Table 1:** Variables used in the analysis and their sources

Variable	Proxy for	Description	Source
FDI_IN_	Insertion in global production networks in terms of inward FDI and trade flows from Europe	Annual average $ of inward FDI *per capita* from Europe during 2003–2014.	FDI markets
FDI_OUT_	Insertion in global production networks in terms of outward FDI and trade flows towards Europe	Annual average $ of outward FDI *per capita* towards Europe during 2003–2014.	FDI markets
Trade	Trade exposure to the EU	Percentage of regional GDP exported to the EU.	Los et al. (2017)
Openness	Degree of international social engagement of UK citizens	Index constructed on the basis of 2 variables, using the standard deviation from the means: 1. UK‐born whose native language is English capable to converse in other language (2001) 2. Log of £ of expenditures per UK inhabitant for outbound overseas trips (2013) *Note*: Data only available for NUTS 2 welsh and English regions and for Scotland as a whole.	British household panel survey (BHPS); national travel survey
UKIP	General consolidate attitude towards the EU	Percentage of UKIP voters at the 2015 national elections	UK electoral commission
Age	Regional demographic structure	Percentage of regional population 20–34 year old (2013).	Eurostat
Educ	Educational attainments	Percentage of employed people holding tertiary education degree (2008‐2014 average).	Eurostat
U	Labour market and economic conditions prior to the Brexit vote	Percentage of regional population claiming JSA unemployment benefits (2008–2014 average).	Nomis dataset
EU funds	Regional investments financed through EU funds	Average annual EU structural fund payments during the 2007–2013 EU programming period per inhabitant.	DG Regional Policy, European Commission
Immig	Migration patterns towards UK regions prior to the Brexit vote	Growth of migrants from outside the UK between 2001 and 2011. *Note*: Only available for Welsh and English regions.	UK census

## 4 RESULTS AND DISCUSSION

### 4.1 The geography of the drivers of the Leave vote: Economic internationalization and cultural openness

Before presenting the results of the OLS estimation of Equation 1, it is worth looking at how our key explanatory variables relate to the Brexit vote and whether or not they corroborate our hypothesis on the role of “split Europeanization.”

**Figure 1:**
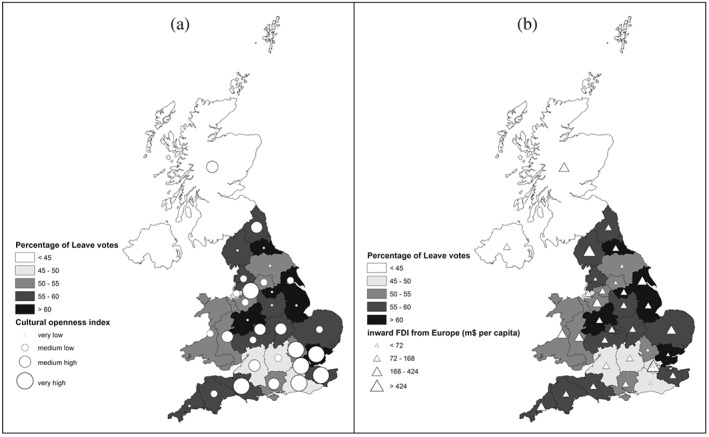
Cultural openness, inward FDI from Europe, and Leave votes: maps of the UK regions *Notes*: Region codes correspond to NUTS 2 codes. Outliers: UKI1 is ‘Inner London’; UKD1 is ‘Cumbria’; UKM0 is ‘Scotland’. The very high proportion of inward FDI recorded in Cumbria is due to a single large investment by Spanish company 'Iberdrola' in 2008 for a total value of $2,565 m. Given that FDI are standardised by population and that Cumbria has only 500,000 inhabitants, this makes it the region with most investment pc. In absolute terms, London has received a much higher investment, $23,828 m in total for 2003–2014. Cumbria received $4,7837 m in the same period.

**Figure 2:**
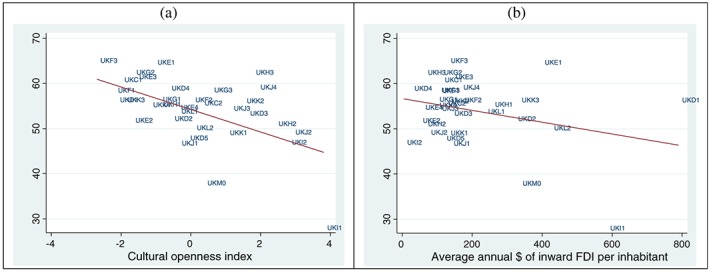
Cultural openness, inward FDI from Europe, and Leave votes: scatterplot correlations *Notes*: Region codes correspond to NUTS 2 codes. Outliers: UKI1 is ‘Inner London’; UKD1 is ‘Cumbria’; UKM0 is ‘Scotland’. The very high proportion of inward FDI recorded in Cumbria is due to a single large investment by Spanish company ‘Iberdrola’ in 2008 for a total value of $2,565 m. Given that FDI are standardised by population and that Cumbria has only 500,000 inhabitants, this makes it the region with most investment pc. In absolute terms, London has received a much higher investment, $23,828 m in total for 2003–2014. Cumbria received $4,7837 m the same period.

Figures 1 and 2 show descriptive maps and scatterplot correlations of UK regions by level of ‘cultural openness’, as defined by our index, the proportion of inward FDI, and the voting outcomes at the 2016 Referendum. Panel A in Figure 1 shows that the highest degree of social/cultural openness is found in the South West of the country, which includes some of the regions where Remain obtained the majority of votes (Inner London, Outer London, Surrey and West Sussex). Although high values of the index are also visible in Essex, where Leave won by a very large margin, on average the relationship between these two variables is negative. As shown by the slope of the regression line in Panel A of Figure 2, higher levels of cultural openness (areas where people were either speaking another language or spending more money to travel abroad) had on average a lower percentage of Leave voters.

Panel B of Figure 1 illustrates the geographical distribution of FDI from European countries to UK NUTS 2 regions, in correspondence with the Referendum results in the same regions. In this case, it is harder to distinguish a clear pattern. Some pro‐Remain regions like London and Scotland received relatively large proportions of foreign investments, but so did pro‐Leave areas such as Cumbria. Panel B of Figure 2 reports a weak negative correlation between these two variables.

### 4.2 Results

Although the bivariate correlations depicted in the scatterplots in subsection 4.1 are informative, they cannot be taken at face value. The effect of all possible determinants of the Brexit vote need to be evaluated jointly to draw more sound conclusions. We do so in our empirical model. Table 2 presents the results of our estimations starting from the first variable of interest – the cultural openness as a proxy for the international engagement of the society, particularly towards Europe 9The fact that many UK citizens speak South Asian or African languages as their second languages may imply that the ‘cultural openness’ index is inaccurate in reflecting the level of ‘Europeanisation’ in the society. To avoid that issue, we replicate the analysis by controlling for the percentage of regional population of Asian and African origin (measured in 2001). The results, available upon request, are not qualitatively different from the ones reported in the paper. – followed by Inward FDI as a proxy for the internationalization of the regional economy and their interaction term. Columns (4) to (10) progressively include the full set of controls. A set of robustness tests are included in the Appendix.

**Table 2:** Internationalization of the society, internationalization of the economy, and Brexit

Dep. Variable: Percentage of Leave votes	(1)	(2)	(3)	(4)	(5)	(6)	(7)	(8)	(9)	(10)	(11)
Cultural openness index	–2.487**	–2.634***	–1.154*	–0.757**	–0.772**	–0.552	–0.568	–0.631*	–0.587	–0.532	–0.354
	(0.968)	(0.749)	(0.635)	(0.320)	(0.319)	(0.316)	(0.336)	(0.327)	(0.355)	(0.334)	(0.323)
Inward European FDI *per capita* (million $)		–0.0155*	–0.0164***	–0.00369	–0.00303	–0.00301*	–0.00292*	–0.00266	–0.00265	–0.00377	–0.00692**
		(0.00843)	(0.00499)	(0.00259)	(0.00269)	(0.00158)	(0.00160)	(0.00175)	(0.00183)	(0.00231)	(0.00302)
**(**inward Eur FDI) x (cultural openness index)			–0.00665***	–0.00362***	–0.00262***	–0.00182**	–0.00174**	–0.00164**	–0.00155*	–0.00168*	–0.00312**
			(0.00136)	(0.000694)	(0.000765)	(0.000657)	(0.000704)	(0.000683)	(0.000688)	(0.000814)	(0.00109)
% votes for UKIP at 2015 elections				1.336***	1.317***	1.222***	1.225***	1.199***	1.176***	1.147***	1.158***
				(0.0524)	(0.0542)	(0.0589)	(0.0560)	(0.0484)	(0.102)	(0.116)	(0.123)
20–34 year old population					–0.217*	–0.672**	–0.735**	–0.827**	–0.799**	–0.697**	–0.849**
					(0.118)	(0.259)	(0.283)	(0.264)	(0.292)	(0.290)	(0.323)
Unemployment benefit claimants						2.129*	2.267**	2.676**	2.263	2.320*	2.678*
						(1.010)	(1.000)	(1.020)	(1.293)	(1.198)	(1.250)
Employed people with tertiary education							0.0216	0.0288	0.0185	0.0137	0.0123
							(0.0417)	(0.0360)	(0.0544)	(0.0453)	(0.0472)
EU funds per inhabitant								–0.0137	–0.0149	–0.0111	–0.0107
								(0.00835)	(0.00911)	(0.00825)	(0.00886)
Growth of migrants from outside the UK									1.489	1.353	2.114
									(3.165)	(3.100)	(3.165)
Percentage of exports towards the EU										0.352	0.432
										(0.392)	(0.391)
Outward European FDI *per capita*											0.00297
											(0.00166)
Constant	54.24***	57.11***	57.12***	35.64***	40.01***	45.67***	45.73***	47.16***	47.26***	42.34***	43.15***
	(1.029)	(1.438)	(1.018)	(1.193)	(2.601)	(3.508)	(3.410)	(3.296)	(3.589)	(7.385)	(7.313)
Observations	33	33	33	33	33	33	33	33	32	32	32
R‐squared	0.312	0.434	0.551	0.925	0.928	0.948	0.948	0.952	0.945	0.947	0.949

*Notes*: Clustered standard errors at NUTS1 level in parentheses.

*** p < 0.01,

** p < 0.05,

* p < 0.1.

The degree of internationalization of the local society – proxied by the cultural openness index – is associated with a statistically significant reduction in the share of voters supporting the UK departure from the European Union (column (1)). This relationship becomes even stronger when the internationalization of the economy is accounted for by adding inward FDI to the model (column (2)). The presence of foreign firms in the local economy also reduces the propensity to vote Leave, although the coefficient is only marginally significant. However, what seems to be the most important factor to shape local preference for EU membership is the interaction between the economic and the social sphere: the interaction term between the cultural openness index and inward FDI (column (3)) is negative and highly significant, suggesting a strong complementarity between these two dimensions. The incidence of Leave votes is minimized in areas where both the local economy and society are jointly internationalized. The two internationalization variables and their interaction term alone can account for more than 50% of the overall regional variability in the EU referendum voting patterns.

The progressive introduction of additional control variables – to be assessed with caution given the limited number of total observations – confirms this overall message. The interaction between social and economic internationalization remains a robust predictor of the share of Leave votes in all specifications, confirm the initial working hypothesis of this paper. Contrary to the initial claims by some commentators the economy does matter to explain voting patterns in the Brexit referendum but it counts the most when interacting with societal attitudes and preferences.

The introduction of additional controls unveils additional interesting features of the geography of the Brexit referendum. First, although the magnitude of the coefficient decreases as more variables are included in the model, the percentage of UKIP voters in 2015 is a strong predictor of the Brexit vote. Obviously this variable picks up some cultural and political values that are unrelated to all the other variables included in the model, such as age, education, but also cultural openness.

Second, as for the demographic variables, age is a strong predictor of the Brexit vote. Even after controlling for all different factors, the effect of age holds strong. This means that if we take two individuals, identical in terms of cultural openness, unemployment, education and so on, living in areas identical in terms of EU funds received, export to the EU and FDIs, the younger individual would still be more likely than the older individual to vote in favour of Europe. This confirms what all the major commentators were reporting in the days after Brexit, despite the fact that they were presenting rather simplistic analyses. However, our proxy for education (i.e., the percentage of workers with tertiary education) is never significant, showing that, once other factors are controlled for, the differential effect of education is negligible.

Third, despite the focus on immigration in the period before the Brexit referendum, the growth of immigrants in an area in the decade 2001–2011, inserted from column (9), is not significantly related to the proportion of Leave votes. The coefficient of the variable is positive, coherently with the existing descriptive literature documenting a positive association between the increase in migrants and the Leave share (Carozzi, 2016; Coyle, 2016; Clark & Whittaker, 2016). However, the inclusion of other factors influencing the vote in our model makes it always insignificant, meaning it did not influence the vote one way or another. This finding contradicts the popular idea that a growth in immigration could spur an anti‐EU sentiment. However, in interpreting this result a deeper reflection is needed. Among their results Harris and Charlton (2017) also found that certain groups such as Pakistanis and Indians were more likely to vote to leave Europe. In the 2016 referendum the most penalized were the European immigrants who never felt the necessity to get a UK citizenship (and hence could not vote). Most immigrants coming from outside the EU eventually took the British citizenship and could therefore vote in the referendum. They might have also played a role in supporting Brexit.

Lastly, areas which receive a larger share of EU structural funds were, indeed, neither more nor less likely to vote for the EU, as shown by the insignificance of the estimated coefficient. The result of an insignificant impact of Cohesion Policy on the Brexit outcome, already reported by other studies (Becker et al., 2016; Crescenzi, Di Cataldo, & Giua, 2017; Fidrmuc, Huényi, & Tunali, 2016), can have multiple explanations. One possibility is that EU subsidies have not contributed to spread pro‐Europe feelings in the most highly funded areas because they have been perceived by some voters as a form of foreign dependence (Davies, 2016). A different interpretation is that EU funds had no influence on voting outcomes simply because large sections of UK voters were entirely unaware of the contribution or scale of EU financial support to their regions. This view is corroborated by the results of a recent EU opinion poll, finding that less than one in ten UK citizens knew about EU‐financed projects in the area they live (European Commission, 2015).

The same is true for trade flows: they received a lot of attention following the Referendum results but they are not robust predictors of the referendum results after carefully controlling for other features of the regional economies. The off‐shoring of domestic activities abroad (outward FDI) is also not significant in the fully specified model. 10When we include outward FDI in the model we may incur in collinearity issues, due to the correlation of this variable with inward FDI. Indeed, as the VIF test is performed (reported in Table A2 in the Appendix), some evidence of collinearity emerges, although the regression results on the key variables of interest remain unchanged when all variables are included (column (11)). The only key economic factors that seems to be correlated with regional leave votes is unemployment. Areas with higher unemployment did vote “Leave” significantly more than others and this result is robust throughout the different model specifications. Economic conditions, at least in terms of unemployment, did matter in defining the outcome of the referendum.

A number of robustness tests are performed in order to confirm the conditional correlations discussed above. The additional regressions are reported in the Appendix. In particular the sign and significance of the key regressors is confirmed after excluding London from the sample. London is considered as a major outlier in the UK (and European economy) in terms of its internationalization (‘global city’) as well as in terms of its economic and social composition and voting patterns (Londoners overwhelmingly voted to remain in the EU). Regressions are also re‐estimated with alternative proxies for FDI, using the number of jobs created rather than the dollar value of the investments as proxy for internationalization (Table A3). This alternative specification confirms the key result of the analysis, that is, the mitigation of anti‐EU feelings in presence of more internationalized people and places.

## 5 CONCLUSIONS

Some commentators have quickly (possibly too quickly) dismissed economic factors as relevant explanations for the success of the Leave vote in many UK regions. Cultural, demographic (age and concentration of foreign migrants) and human capital (education) explanations have dominated the debate, often relegating pro‐Brexit support to ageing and poorly educated groups and areas. Conversely, economic narratives have pointed their attention toward the discontent generated by the process of globalization via international trade flows. This paper has taken a different approach by exploring the middle ground between economic and cultural explanations and looking at the interaction between economic and social factors as the most promising avenue to answer the question “why has this happened?”

First, the paper has developed more comprehensive proxies for the degree of economic and cultural integration of the UK regions. The former has been captured by taking into account the role of FDI: while free trade is largely possible under WTO rules, full capital mobility is indeed a feature of the Single Market. The latter has been captured by means of a ‘cultural openness’ index hat captures the cultural internationalization of British voters in a way that is independent from migration inflows. Second, our work has explored the tension between the internationalization of the regional economy and the internationalization of the underlying society as a possible predictor of the propensity to support the UK departure from the European Union. The robustness of this possible interpretation of the electoral data has been tested against alternative hypotheses developed in the media and academic debate on the topic.

The basic analysis of the correlation between Leave votes and internationalization proxies suggests that both exposure to FDI and cultural/social openness reduce the share of anti‐EU votes. However, the stronger predictor for Euroscepticism is the interaction between these two dimensions: regions where the presence of foreign firms is not coupled by the propensity of local workers to interact with foreign cultures are more likely to vote “Leave.” In other words, if internationalization in the workplace is not coupled by internationalization “at home,” it tends to increase the pressure on local workers to vote out from further economic integration. This explanation of the geography of the Brexit vote is robust to the inclusion of a number of controls as proxies for other influencing factors. The propensity of elderly voters to support Brexit is confirmed by our analysis, together with the share of UKIP votes in the previous political elections as a measure for *a priori* anti‐EU sentiments in the local population. Unemployment is the single most important economic factor correlating with Leave votes, on top of our measures for internationalization.

The analysis presented in the paper suffers from a number of limitations. The EU Referendum is a one‐off event that makes it impossible to include any time dimension to the analysis. This makes ‘unobservable’ factors a significant challenge to any quantitative analysis of the electoral data. Even if the empirical model takes into account a large number of variables – mitigating this potential problem – the limited number of observations (all UK NUTS 2 English and Welsh regions, plus Scotland) inevitably constraints the number of regressors. In addition the results can only be interpreted as conditional correlations without any causal message. In addition our hypothesis about split Europeanization would be better tested by means of micro‐level individual employer‐employee matched data that would make it possible to exactly link the workplace internationalization of each individual with their cultural orientation. However, unfortunately, these data are very hard to obtain and – as the unpredictability of the referendum results has shown – difficult to link to actual voting decisions (for example vs. responses to hypothetical questions on attitudes towards the EU).

Having acknowledged these limitations, the paper still offers relevant descriptive insights on the localized factors behind the Brexit vote. It offers – together with other contributions in this emerging stream of political economy research – material for discussion on the lessons to be learnt from the UK experience on how to ensure the political sustainability of the process of European integration. Dismissing economic factors behind anti‐EU voting patterns can lead to misleading actions and policies. Inclusive labour markets remain important conditions for an equitable distribution of the benefits from the process of integration. However, even in high‐employment countries like the UK, the intensified role of global investment flows and value chains in the economic tissue of EU countries and regions – in particular in the form of the increased presence of foreign firms operating across national borders – poses major societal challenges. If social and cultural attitudes towards integration and internationalization are not developed in parallel with the globalization of the economy, tensions are very likely to arise.

## ACKNOWLEDGEMENT

The research leading to these results has received funding from the European Research Council under the European Union Horizon 2020 Programme H2020/2014‐2020 (Grant Agreement n 639633‐MASSIVE‐ERC‐2014‐STG). We would like to thank Phil McCann and two anonymous referees for very helpful comments that helped to shape our ideas. Feedback from participants in the RSAI‐BIS Conference and the RSA Winter Conference in London is also gratefully acknowledged. All errors remain our own.

## APPENDIX 1

### 1.1

**Table A1:** Descriptive statistics

Variable	Obs	Mean	Std. dev.
Percentage of leave votes	33	54.247	7.413
Inward European FDI *per capita* (million $)	33	185.5	168.1
Cultural openness index components:			
Capacity to converse in other language	33	0.237	0.089
Log £ for outbound trips *per capita*	33	4.891	1.280
Cultural openness index	33	–0.002	1.665
Percentage of votes for UKIP at 2015 elections	33	14.367	4.005
Percentage of 20‐34 year old population	33	19.287	3.219
Employed people with tertiary education	33	37.225	9.848
Percentage of unemployment benefit claimants	33	2.106	0.682
Growth of migrants from outside the UK (2001–2011)	32	0.663	0.265
Annual average EU funds per inhabitant (2007–2013)	33	32.702	39.974
Percentage of exports towards the EU	33	10.245	1.495
Outward European FDI *per capita* (2003–2014)	33	148.1	411.2
Inward European FDI jobs created *per capita*	33	0.339	0.283

**Table A2:** VIF test

	With outward FDI (column (11))		Without outward FDI (column (10))	
**Variable**	VIF	1/VIF	VIF	1/VIF
Cultural openness index	3.35	0.298	2.35	0.425
% votes for UKIP at 2015 elections	1.9	0.527	1.88	0.532
Percentage of 20–34 year old population	11.52	0.086	8.78	0.113
Employed people with tertiary education	2.55	0.392	2.54	0.393
Percentage of unemployment benefit claimants	4.91	0.203	4.22	0.236
Growth of migrants from outside the UK	2.84	0.351	2.39	0.418
Annual average EU funds per inhabitant	1.52	0.658	1.52	0.659
Percentage of exports towards the EU	2.49	0.402	2.33	0.429
Inward European FDI *per capita* (million $)	4.72	0.211	1.59	0.627
Outward European FDI *per capita*	17.08	0.058		
(inward Eur FDI) x (cultural openness index)	10.71	0.093	4.01	0.249
Mean VIF	5.78		3.16	

**Table A3:** Robustness tests – Inner London excluded

Dep. Variable: Percentage of leave votes	(1)	(2)
Cultural openness index	–0.103	–0.106
	(0.379)	(0.535)
Inward European FDI *per capita* (million $)	–0.0112**	
	(0.00469)	
(inward Eur FDI) × (cultural openness index)	–0.00519**	
	(0.00176)	
% votes for UKIP at 2015 elections	1.121***	1.073***
	(0.0996)	(0.121)
20–34 year old population	–1.077***	–0.835**
	(0.294)	(0.336)
Unemployment benefit claimants	3.093**	2.808**
	(1.296)	(1.114)
Employed people with tertiary education	0.0295	–0.00123
	(0.0406)	(0.0526)
EU funds per inhabitant	–0.0143	–0.0156
	(0.00826)	(0.00964)
Growth of migrants from outside the UK	1.922	0.950
	(2.765)	(3.408)
Percentage of exports towards the EU	0.389	0.414*
	(0.327)	(0.206)
Outward European FDI *per capita*	–0.00114	–0.00218
	(0.00171)	(0.00412)
Inward European FDI jobs created *per capita*		–3.663
		(2.068)
(inward Eur FDI jobs) × (cultural openness index)		–3.081*
		(1.425)
Constant	48.03***	45.81***
	(6.009)	(7.009)
Observations	31	31
R‐squared	0.918	0.927

*Notes*: Clustered standard errors at NUTS1 level in parentheses.

*** p < 0.01, **p < 0.05, *p < 0.1. Inner London observation excluded in both specifications.
